# Using presence-only and presence–absence data to estimate the current and potential distributions of established invasive species

**DOI:** 10.1111/j.1365-2664.2010.01911.x

**Published:** 2011-02

**Authors:** Andrew M Gormley, David M Forsyth, Peter Griffioen, Michael Lindeman, David SL Ramsey, Michael P Scroggie, Luke Woodford

**Affiliations:** 1 Arthur Rylah Institute for Environmental Research, Department of Sustainability and Environment123 Brown Street, Heidelberg 3084, Australia; 2 Peter Griffioen Consulting3/391 Upper Heidelberg Road, Ivanhoe 3079, Australia

**Keywords:** camera trap, *Cervus unicolor*, detection probability, habitat suitability models, kernel smoothing, Maxent, occupancy, sambar deer, state-space modelling, Victoria

## Abstract

**1.**Predicting the current and potential distributions of established invasive species is critical for evaluating management options, but methods for differentiating these distributions have received little attention. In particular, there is uncertainty among invasive species managers about the value of information from incidental sightings compared to data from designed field surveys. This study compares the two approaches, and develops a unifying framework, using the case of invasive sambar deer *Cervus unicolor* in Victoria, Australia.

**2.**We first used 391 incidental sightings of sambar deer and 12 biophysical variables to construct a presence-only habitat suitability model using Maxent. We then used that model to stratify field sampling, with proportionately greater sampling of cells with high predicted habitat suitability. Field sampling, consisting of faecal pellet surveys, sign surveys and camera trapping, was conducted in 80 4-km^2^ grid cells. A Bayesian state-space occupancy model was used to predict probability of suitable habitat from the field data.

**3.**The Maxent and occupancy models predicted similar spatial distributions of habitat suitability for sambar deer in Victoria and there was a strong positive correlation between the rankings of cells by the two approaches. The congruence of the two models suggests that any spatial and detection biases in the presence-only data were relatively unimportant in our study.

**4.**We predicted the extent of suitable habitat from the occupancy model using a threshold that gave a false negative error rate of 0·05. The current distribution was the suitable habitat within a kernel that had a 99·5% chance of including the presence locations pooled from incidental sightings and field surveys: the potential distribution was suitable habitat outside that kernel. Several discrete areas of potential distribution were identified as priorities for surveillance monitoring with the aim of detecting and managing incursions of sambar deer.

**5.***Synthesis and applications.*Our framework enables managers to robustly estimate the current and potential distributions of established invasive species using either presence-only and/or presence–absence data. Managers can then focus control and/or containment actions within the current distribution and establish surveillance monitoring to detect incursions within the potential distribution.

## Introduction

Invasive species can have important detrimental environmental, economic and social impacts (Mack *et al.* 2000; Pimentel *et al.* 2005; Lodge *et al.* 2006) and there is much interest in managing these populations (Myers *et al.* 2000; Hulme 2006; Lodge *et al.* 2006). Predicting and quantifying the current and potential distributions of established invasive species is a critical step in evaluating management options: for example, control and eradication efforts should focus on the current distribution, containment should focus on the interface between the current and potential distributions, and incursion monitoring should focus on the potential distribution (Myers *et al.* 2000; Leung *et al.* 2005; Lodge *et al.* 2006). However, methods for differentiating the current and potential distributions of established invasive species have received little attention.

Since the distributions of many established invasive plants and animals may be much smaller than their maximum distributions (e.g. for recent and/or slow invaders; Ward 2007; Phillips, Chipperfield & Kearney 2008), methods are required for discriminating suitable habitat that is occupied from that which is unoccupied. The first step is to distinguish ‘suitable’ from ‘unsuitable’ habitat, and two general approaches have been used to do this. Presence-only data (e.g. from atlas records) and biophysical variables can be used to fit predictive ‘niche-based models’ of distribution using numerous methods (Elith *et al.* 2006). Models of presence-only data produce spatially explicit suitability surfaces that represent habitat suitability (Elith *et al.* 2006). However, presence-only modelling based on incidental sightings may be subject to major spatial and detection biases (Gu & Swihart 2004; Wintle, Elith & Potts 2005; Araújo & Guisan 2006). An alternative approach is to conduct field surveys in a way that accounts for potential spatial biases (by using a known sampling design; Thompson, White & Gowan 1998) and imperfect detection of the species of interest (MacKenzie *et al.* 2002): modelling such data estimates the probability of occupancy (MacKenzie *et al.* 2006). Occupancy models constructed from observed presence–absence data also predict habitat suitability when projected across the landscape. A threshold is needed to distinguish the output of habitat suitability models (from presence-only and presence–absence models) into ‘suitable’ and ‘unsuitable’ habitat (Liu *et al.* 2005).

The second step is to estimate which areas of predicted suitable habitat are ‘occupied’ (‘current distribution’) and ‘unoccupied’ (‘potential distribution’). Point pattern analysis (‘kernel smoothing’; Diggle 2003; Hengl *et al.* 2009) is a particularly promising method for estimating the current distributions of established invasive species because it can use presences pooled from presence-only and presence–absence data.

The aim of this study is to estimate the current and potential distributions of invasive sambar deer *C. unicolor* Kerr in the state of Victoria, Australia. We first construct habitat suitability models for sambar deer using presence-only data from incidental sightings and presence–absence data from a designed field survey. After comparing the predictions of the two methods we then use threshold occupancy and kernel smoothing methods to delineate the current and potential distributions of sambar deer in Victoria.

## Materials and methods

### Study area and species

**Fig. 1:**
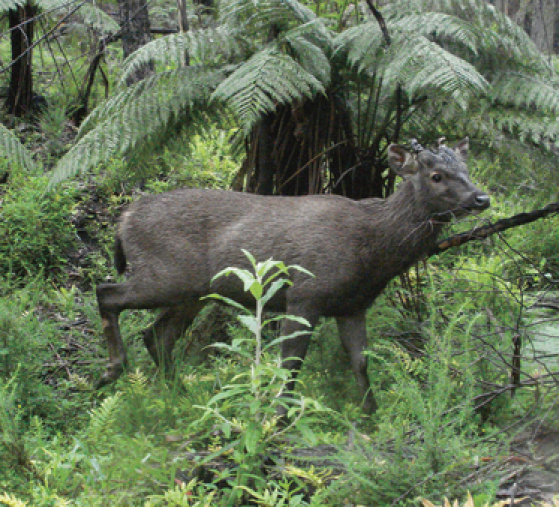
Sambar deer photographed at a camera trap during our presence–absence field survey.

The state of Victoria (237 629 km^2^), south-eastern mainland Australia, was our study area. Sambar deer (Fig. 1), sourced from Sri Lanka, India and the Philippines, were introduced at four sites in Victoria during the 1860s and have subsequently expanded their distribution to the north, north-east and south-east of Victoria (Menkhorst 1995; Bentley 1998). There is concern about the continued range expansion of sambar deer in Victoria because of their potential negative impacts on native biodiversity (Department of Sustainability and Environment 2009a) and agriculture (Lindeman & Forsyth 2008).

We subdivided Victoria into 56 764 cells of 2 × 2 km. A cell size of 4 km^2^ was chosen because it approximated estimates of sambar deer home range size in invasive populations (Lewis *et al.* 1990; Fraser & Nugent 2005) and was a practical unit size for conducting field surveys (*sensu*Karanth *et al.* 2009).

### Predictor variables

Thirty biophysical variables were identified from the literature as potentially important predictors of sambar deer distribution and abundance in Victoria (review in Forsyth *et al.* 2009; see Appendix S1, Supporting information). The variables were generated, for each of the 4 km^2^ cells, from GIS layers supplied by the Victorian State Government’s Corporate Geospatial Data Library (O’Brien 2004). Prior to model building we assessed the strength of Pearson’s correlation coefficients between pairs of variables: if variables were highly correlated (*r*_p_ > 0·7) then one of the variables was removed from the set. A final set of 12 candidate variables remained for model building (Table 1).

**Table 1:** Biophysical covariates used in our models of sambar deer distribution in Victoria

Covariate	Description	Units
Grass	Amount of grassland	% (0–100)
Gullies	Number of gullies	Count
Homogeneity	Similarity of land use	0–100
NativeGrassShrub	Amount of native grassland/shrubland	% (0–100)
AnnualPrecip	Annual precipitation	mm
SeasonalPrecip	Seasonal difference in precipitation	mm
RoadDistance	Distance from nearest road	m
MeanTemp	Annual mean temperature	°C × 10
MinimumTemp	Minimum annual temperature	°C × 10
WaterDistance	Distance from water	m
WetForestCover	Amount of wet sclerophyll forest	% (0–100)
Slope	Average slope	° (0–90)

### Habitat suitability model from incidental sightings

#### Incidental sightings

Presence-only data for sambar deer were obtained from the Atlas of Victorian Wildlife Database (AVWD) containing data from 1974 to 2007 (Department of Sustainability and Environment 2009b). The AVWD is a geographically registered relational database of incidental sightings of fauna by government agency staff and the public. Sambar deer observations consisted of a date, latitude/longitude and a measure of locational precision. We only used records (*n* = 391) with a location precision of less than 1 km in our analyses, and binned these observations into the 4 km^2^ cells.

#### Maxent model

Incidental sightings of sambar deer were modelled using Maxent 3·2·19 (Phillips, Anderson & Schapire 2006), a machine learning approach based on maximum entropy. Maxent has been shown to perform as well as, or better than, other methods for modelling presence-only data (Elith *et al.* 2006). Maxent uses the presence-only data and a user-defined number (in our case, 10 000) of randomly selected points (‘pseudo-absences’) and combines these with the biophysical covariates to construct an index of habitat suitability for each cell ranging from 0 (least suitable habitat) to 1 (most suitable habitat). We allowed linear and/or quadratic relationships between the index of habitat suitability and each covariate (Phillips & Dudík 2008). The relative contribution of each covariate to the Maxent distribution, and the relationship between each variable and the predicted index of habitat suitability, was also calculated (Phillips, Anderson & Schapire 2006).

Model performance was assessed by determining how well the model discriminates between unsuitable and suitable habitat over a range of thresholds (Fielding & Bell 1997). For any threshold of habitat suitability index, presence locations are either correctly classified as being in suitable habitat (‘true positives’) or misclassified as being in unsuitable habitat (‘false negatives’). Similarly, absence data are either correctly classified as being in unsuitable habitat (‘true negatives’) or misclassified as being in suitable habitat (‘false positives’). Because false positives cannot be estimated for presence-only data, Maxent estimates the fractional predicted area (FPA), which is the proportion of cells predicted to have suitable habitat for the species (Phillips, Anderson & Schapire 2006). To assess performance of the Maxent model we plotted a receiver operating characteristic curve, which compares the model sensitivity (true positives) against 1 – specificity (false positives) over the entire range of thresholds (Fielding & Bell 1997). For presence-only modelling, the area under this curve (AUC) represents the probability that a randomly chosen presence site will be ranked as more suitable than a randomly chosen pseudo-absence site. A model that performs no better than random will have an AUC of 0·5 whereas a model with perfect discrimination would have an AUC of 1. An additional measure of model performance is the regularized training gain (‘Gain’), which describes how much better the Maxent distribution fits the presence data compared to a uniform distribution. The exponential of the Gain is a measure of how many times higher the sample likelihood is compared to a random cell (Yost *et al.* 2008).

### Occupancy model from field surveys

#### Sampling methodology

Our aim here was to develop a model of potential distribution of sambar deer based on the relationship between presence/absence data and biophysical variables. Since resources were available to conduct field surveys in only 80 cells, it was desirable to spend more effort sampling areas of high-habitat suitability (*sensu*McDonald 2004). We therefore allocated a greater proportion of sites to areas of higher habitat suitability estimated by our Maxent model. Sixty cells were randomly selected and retained with probability equal to the corresponding habitat suitability index of that cell. The other 20 cells were selected entirely at random.

#### Field surveys

We used three survey methods to estimate occupancy rates of sambar deer between July 2008 and April 2009. First, we assessed presence/absence of sambar deer faecal pellets along three randomly located transects in each of the 80 cells using the method described in Forsyth *et al.* (2007). Briefly, we navigated to the start of each 150-m transect using a hand-held GPS and counted the number of intact pellets in circular plots of 1 m radius spaced at 5 m intervals (i.e. 30 plots per transect). The presence and absence of pellets in cell *i* and transect *j* was indicated by *Y*_*ij*_ = 1 and 0, respectively, for *j* = 1–3.

Secondly, we searched for signs of sambar deer along a 400 m transect in each of the 80 cells. The sign transect was subjectively located by field staff to maximize the detection of deer (e.g. along a trail or watercourse likely to be used by sambar deer; Bentley 1998). Any of the following signs of sambar deer seen along the survey route were recorded: sightings of live or dead deer, tree-rubbings, tracks, cast antlers, wallows and faecal pellets. The presence/absence of sambar deer sign on transects was denoted as *Y*_*i*4_ = 1 and 0, respectively.

Thirdly, in a randomly selected 40 of the 80 cells we set two heat-in-motion remote cameras along the sign survey route. Cameras [TrailMAC Digital (Trail Sense Engineering, Middletown, DE, USA) and PixController DigitalEye™ (PixController Inc., Export, PA, USA)] were set, unbaited, for 21 days. The presence/absence of images of sambar deer on the cameras was indicated by *Y*_*i*5_ = 1 and 0, respectively.

#### Statistical model

The presence–absence data were modelled using a Bayesian state-space occupancy model consisting of a process model and an observation model (Royle & Kéry 2007). The process model describes the true occupancy at each site and the observation model described the observation process conditional on the true occupancy state of each site. For each site *i*, the true occupancy state *z*_*i*_ was modelled as a random variate from a Bernoulli distribution with probability *ψ*_*i*_ equal to the probability of occupancy at site *i*: 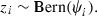
eqn 1

The probability of occupancy at site *i* was modelled as a function of one or more biophysical covariates, denoted in general as: 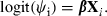
eqn 2

For each survey method there is a probability of detection given that the site is occupied. The observed presences/absences were modelled as: 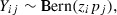
eqn 3 where *Y*_*ij*_ is the observed presence/absence at site *i* for survey *j*, and *p*_*j*_ is the detection probability for that survey (recall *j* = 1–3 denotes faecal pellet transects, *j* = 4 sign surveys and *j* = 5 camera surveys). If a site is unoccupied then *z*_*i*_ = 0 and *Y*_*ij*_ = 0 is observed with probability 1. If a site is occupied then *z*_*i*_ = 1 and *Y*_*ij*_ = 1 is observed with probability *p*_*j*_, and *Y*_*ij*_ = 0 with probability 1–*p*_*j*_. Assuming independence of the surveys, the overall probability of detection, conditional on presence, p*, from *k* surveys is: 
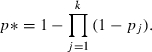
eqn 4

The same twelve biophysical variables used in the Maxent model (Table 1) were used as potential covariates in the occupancy model.

#### Parameter estimation

Models were fitted using WinBUGS 1·4·3 (Lunn *et al.* 2000). Prior distributions of Normal(0, 100) were used for the covariate coefficient parameters ***β***. All covariates were standardized to a mean of 0 and standard deviation of 1. Prior distributions of Beta(1, 1) were used for the detection probabilities *p*_*j*_ for each of the three survey methods. Three replicate Markov-chains were constructed using different initial values to check for convergence. The chains were run for 1000 iterations to tune the algorithm and ensure convergence. The ‘burn-in’ samples were discarded and the algorithm run for a further 20 000 samples before the three chains were combined to provide a sample of 60 000 values from the joint posterior distribution of each parameter. Our WinBUGS code is provided in Appendix S2 (Supporting information).

#### Model selection and averaging

We calculated the deviance information criterion (DIC) value for each model following Spiegelhalter *et al.* (2002). We first evaluated models containing the 12 biophysical variables individually and in pairs. This was followed by models with combinations of three and four variables, using variables that had consistently lower DIC values as individuals and pairs. Rather than selecting a single ‘best’ model we used model averaging (Burnham & Anderson 2002; McCarthy 2007) to predict sambar deer occupancy. Model weights (*w*) were summed from largest to the smallest, and the models with a cumulative sum of 0·9 used as the model averaging set (Burnham & Anderson 2002). The resulting model-averaged predictive equation was applied to each 4 km^2^ grid cell in our study area to produce a map of predicted probability of suitable habitat for sambar deer.

### Comparing predictions of the Maxent and occupancy models

Although Maxent and occupancy models both give results on the unit scale, these are not directly comparable. We therefore compared the predictions (i.e. cell rankings from lowest to highest) of the presence-only Maxent model and the presence–absence occupancy model using Spearman’s correlation coefficient (*r*_s_). We also compared the spatial output from each of the two models following rescaling as deciles.

### Defining ‘suitable habitat’

The probabilities of suitable habitat for each cell from the occupancy model were delineated into suitable and unsuitable habitat using a threshold. The choice of a threshold depends on whether one wishes to minimize false negative or false positive errors, or balance them in some other way (Liu *et al.* 2005). A threshold that is too high will result in a high number of false negative errors and low number of false positives, leading to a higher proportion of the study area being classified as unsuitable when it is suitable. Conversely, a threshold that is too low will result in lower false negative and higher false positive error rates, leading to a relatively high proportion of the study area being classified as suitable when it is not (Ward 2007). We selected a threshold by setting the false negative error rate at 0·05.

### Estimating current distribution

We delimited the current distribution of sambar deer, conditional on areas of suitable habitat, by two-dimensional kernel smoothing the pooled sambar deer presence data (i.e. using both incidental sightings and field survey data). The function ‘kde2d’ in R package ‘MASS’ version 7·2 (Venables & Ripley 2002) with a bivariate Gaussian kernel was used to estimate the density surface. This method has been widely used to estimate the utilization distribution of individual animals based on location data. The resulting density surface can be thought of as indicating the relative intensity (i.e. points per unit area) of species presence records for any location within the study area. The bandwidth for smoothing was calculated using the ‘solve-the-equation’ method of Sheather & Jones (1991) and we defined a percentage level that ensured 99·5% of the presence records were included in the current distribution. Kernel smoothing was applied conditional on the cell being classified as suitable habitat (see above).

## Results

### Habitat suitability model using incidental sightings

**Fig. 2:**
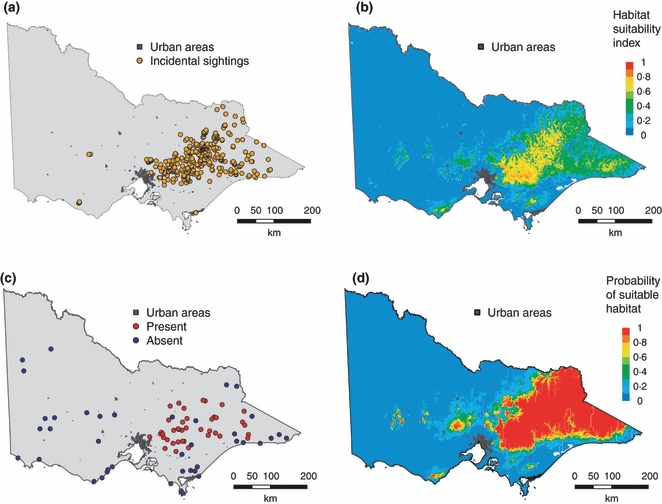
Habitat suitability and occupancy models for sambar deer in Victoria estimated from incidental sighting (presence-only) and field survey (presence–absence) data, respectively. (a) Incidental sightings used in the Maxent habitat suitability model. (b) Predictions of the Maxent habitat suitability model. (c) The 804-km^2^ cells in which presence–absence field surveys were undertaken. (d) Predictions of the occupancy model.

**Fig. 3:**
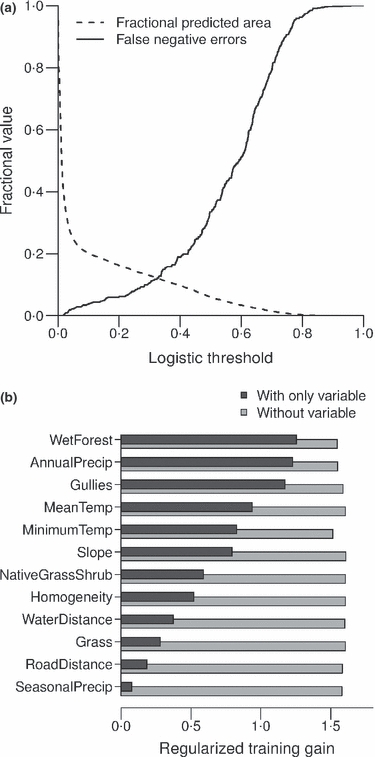
Performance of the Maxent habitat suitability model of incidental sightings of sambar deer in Victoria. (a) False negative error rate (solid line) and FPA (dashed line) for all threshold values. (b) Regularized Training Gain, with variables ranked depending on the Gain from a model with only that variable.

The 391 sightings of sambar deer occurred in 322 cells (Fig. 2a). The AUC (0·942) and Gain (1·61) values indicate that the Maxent model of the incidental sightings (Table 2) had a high discriminatory ability (Fig. 2b). The plot of false negative errors and FPA (Fig. 3a) showed little overlap, further confirming the usefulness of the Maxent model. Three variables (WetForestCover, AnnualPrecip and Gullies) had a relative contribution of 83% to the Maxent model and when used on their own showed a reasonable fit to the data in terms of Gain (Fig. 3b). Conversely, the variables SeasonalPrecip and RoadDistance achieved little Gain when used alone (Fig. 3b). Results from omitting each variable whilst including all others showed that no one variable contained a substantial amount of information that was not contained in the other variables. Three other variables (MeanTemp, MinimumTemp and Slope) showed a reasonable to fit to the data in terms of Gain when used alone despite having small relative contributions to the model built using all variables. The probability of presence increased with increasing WetForestCover and AnnualPrecip, but had a concave-up relationship with Gullies and MeanTemp (Fig. S1, Supporting information).

**Table 2:** Relative contribution of variables to the Maxent model of incidental sightings of sambar deer in Victoria

Variable	Relative contribution
WetForestCover	65·4
AnnualPrecip	9·4
Gullies	8·4
WaterDistance	6·7
MinimumTemp	5·5
RoadDistance	1·7
SeasonalPrecip	1·2
NativeGrassShrub	0·6
Homogeneity	0·5
Grass	0·3
MeanTemp	0·2
Slope	0·1

### Detection probabilities and occupancy model

**Fig. 4:**
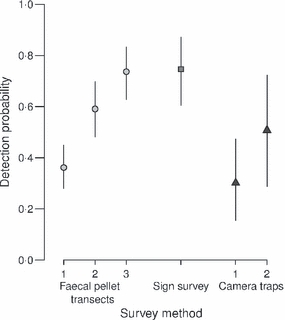
Conditional probabilities of detection for our three field survey methods. Cumulative probabilities are shown for one, two or three faecal pellet transects, and one and two camera traps. Vertical bars are 95% credible intervals.

Sambar deer were detected in 40 of the 80 sampled cells (Fig. 2c). They were detected on one or more faecal pellet transects in 26 cells, on sign transects in 35 cells and at camera traps (Fig. 1) in 10 of the 40 cells sampled with that method. The highest probability of detection, conditional on presence, was associated with sign surveys, followed by transects and cameras (Table 3 and Fig. 4). The overall probability of detection from three faecal pellet transects was 0·736 (95% CI = 0·628–0·832), and from two cameras was 0·507 (95% CI = 0·288–0·722). The site-level detection probability, combining all methods (eqn 4), was 0·932 (95% CI = 0·851–0·974) at sites where only faecal pellet transects and sign surveys were used and 0·967 (95% CI = 0·896–0·992) at sites where all three methods were used.

**Table 3:** Model-averaged parameter estimates from occupancy models of sambar deer in Victoria. SD is the square root of the unconditional variance estimator. Importance is calculated for *β* coefficients as the sum of the model weights for models containing that parameter

Parameter	Mean	SD	2·5%	97·5%	Importance
*α* [Intercept]	1·062	1·172	−0·809	3·370	NA
*β* [Gullies]	0·389	0·638	−0·081	1·119	0·37
*β* [Homogeneity]	0·197	0·409	−0·123	0·648	0·21
*β* [AnnualPrecip]	2·355	1·100	0·595	4·659	0·99
*β* [MeanTemp]	0·723	1·299	−0·549	1·970	0·40
*β* [MinimumTemp]	−4·263	1·573	−7·699	−1·664	1·00
*β* [WetForestCover]	−0·025	0·194	−0·336	0·296	0·21
*β* [Slope]	0·145	0·306	−0·051	0·586	0·26
*p* [Faecal Pellet Transect]	0·362	0·043	0·281	0·449	NA
*p* [Sign]	0·746	0·068	0·605	0·870	NA
*p* [Camera]	0·302	0·815	0·156	0·479	NA

The variables Gullies, Homogeneity, AnnualPrecip, AnnualTemp, MinimumTemp, WetForestCover and Slope had consistently lower DIC values relative to the other covariates when used alone and when included in pairs. Subsequently, all three-way and four-way combinations of these seven covariates were modelled. A total of 148 models with various combinations of covariates were evaluated. The best model (i.e. lowest DIC) included the variables Gullies, AnnualPrecip, AnnualTemp, and MinimumTemp (Table S1, Supporting information). However, there were many models with similar DIC values: the 17 highest ranked models had a cumulative model selection weight of 0·906. The variables MinimumTemp and AnnualPrecip were included in 17 and 16 of the reduced set of 17 models used for model averaging, respectively (Tables S1, Supporting information and 3). There was a strong negative effect of MinimumTemp, and a strong positive effect of AnnualPrecip, on probability of occupancy (Table 3). The effects of the other variables included in the model-averaged occupancy model were more equivocal (Table 3).

### Comparison of the Maxent and occupancy models

**Fig. 5:**
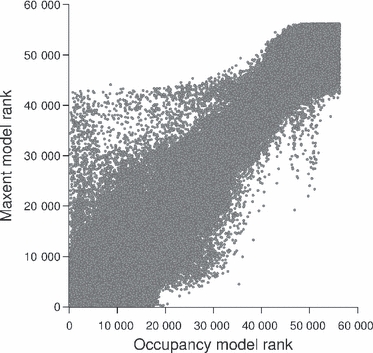
Scatter plot of cell ranks from the predictions of Maxent and occupancy models.

There was a strong positive correlation (*r*_s_ = 0·89) between the rankings of cells by the two methods (Fig. 5): cells with a higher habitat suitability index from Maxent had higher probabilities of suitable habitat from the occupancy model. Both models indicated that areas of highest habitat suitability for sambar deer were in eastern Victoria and that the northern, western and southern areas of the state were of lowest suitability (Fig. 2b,d). There were several large patches of moderate habitat suitability in central and southern Victoria.

Comparison of each cell’s deciles showed several coastal areas were ranked higher in the Maxent model than the occupancy model, although they were still ranked relatively low overall (Fig. S2, Supporting information).

### Current and potential distributions of sambar deer in Victoria

**Fig. 6:**
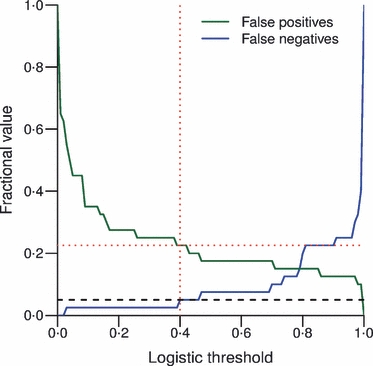
False negative and false positive error rates from the occupancy model for all threshold values. The black dashed line indicates a 5% false negative error rate and the red lines indicate the resulting threshold between suitable and unsuitable habitat and the corresponding false positive error rate for that threshold.

We used all presence-only data (i.e. including all incidental sighting records from 1974–2007 and our field survey presences) to estimate current distribution. The target false negative rate of 0·05 was achieved at a threshold level of 0·40, which had a corresponding commission error rate of 0·225 (Fig. 6). The threshold value of 0·40 was therefore used to delineate between unsuitable and suitable sambar deer habitat. Using this threshold there are an estimated 58 340 km^2^ of suitable sambar deer habitat in Victoria.

**Fig. 7:**
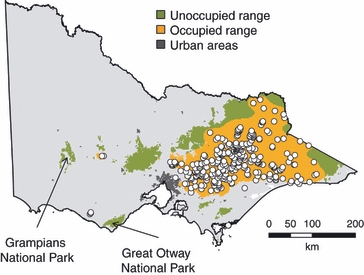
Occupied (orange) and unoccupied (green) ranges of sambar deer in Victoria from an occupancy model and kernel smoothing of presence locations (circles) from incidental sightings and field surveys.

The 99·5% utilization distribution gave a current estimated distribution of 42 888 km^2^ (Fig. 7). Major areas of apparently suitable but unoccupied range outside the current distribution include the Great Otway National Park and Grampians National Park, both in western Victoria (Fig. 7).

## Discussion

We used presence-only (incidental sightings) and presence–absence (field surveys) data to differentiate the current and potential distributions of invasive sambar deer in Victoria such that potentially important spatial and detection biases were minimized. We first used incidental sightings to estimate a habitat suitability index. We then used the habitat suitability index to stratify our field survey effort and our field surveys used methods that enabled imperfect detection to be accounted for in the estimated probability of suitable habitat. We then used a threshold to delineate the predictions of the occupancy model into suitable (i.e. potential distribution) and unsuitable habitat. Finally, we applied kernel smoothing to the pooled presence data (i.e. from both incidental sightings and field surveys) to further delineate the suitable habitat into estimates of current and potential range. Our analyses indicated that sambar deer occupied c. 74% of suitable habitat (42 888 km^2^) in Victoria in 2008–2009 but that several large, discrete areas of potential range exist in western Victoria.

### Congruence of habitat suitability and occupancy models

Although the units of Maxent and occupancy models differ, there was strong agreement between the relative rankings of the predictions of the two approaches for sambar deer in Victoria (Fig. 5). To our knowledge, this is the first study to use independently collected presence–absence data to test the predictions of a habitat suitability model constructed from presence-only data: previous comparisons have used pseudo-absences (e.g. Elith *et al.* 2006; Pearson *et al.* 2007). The congruence of the two models suggests that any spatial and detection biases in the presence-only data (Gu & Swihart 2004; Wintle, Elith & Potts 2005; Araújo & Guisan 2006) were unimportant in our case study. The combination of repeated surveys and multiple field methods when collecting the presence–absence data resulted in a high cumulative detection probability and thus a very small probability of false negatives. However, such biases might be more important for a recently established invader with a small current range and/or few sightings, or when unmodelled processes constrain range expansion (Pearson *et al.* 2007). Furthermore, issues related to detectability are likely to be greater for rare and/or elusive species. MacKenzie *et al.* (2006) give an excellent summary of the logic for using occupancy estimated from designed field surveys, rather than habitat suitability derived from incidental sightings, to estimate species distributions.

We chose to use Maxent to model incidental sightings of sambar deer in Victoria, but many other methods are available for modelling presence-only data (Elith *et al.* 2006). In the absence of any presence-only data, expert opinion could be used to develop a habitat suitability model (e.g. Yamada *et al.* 2003; Ray & Burgman 2006) for stratifying field sampling. However, the ability of experts to extrapolate beyond their geographic area of expertise may be poor (Murray *et al.* 2009).

An alternative to choosing among different modelling approaches when estimating species distributions is to combine inferences using the ensemble model framework (Araújo & New 2007). Future studies could use that framework to combine the outputs of presence-only (e.g. Maxent) and presence–absence models.

### Stratifying field sampling using habitat suitability models

Field surveys are expensive and occupancy rate will be estimated more precisely if proportionately more sampling is conducted in areas where a species is known or predicted to occur relative to areas where they have not previously been observed. We chose to randomly allocate 75% of our field surveys to cells using an unequal probability sampling scheme according to the habitat suitability index and the remainder randomly to all cells. The choice of how to stratify field sampling is determined by the goal of the study. The aim of our study was to estimate current and potential distribution. If the aim was to detect new incursions/range expansions then relatively more effort should be placed in areas of lower habitat suitability. Further work is required to generate rules of thumb for the allocation of survey effort based on habitat suitability maps, and adaptive sampling may be a useful approach (Thompson, White & Gowan 1998).

### Detection probabilities

Although all three field survey methods had detection probabilities <1 (Fig. 4), the use of multiple methods and spatial replication of two of those methods (faecal pellet transects and camera traps) reduced the overall probability of false negatives in sampled cells. Sambar deer are cryptic, being largely nocturnal and spending daylight hours in dense forest (Bentley 1998). If multiple methods were not used, the rate of false negatives would have been much greater. These results highlight the need to carefully consider detection probability in the design of presence–absence surveys (MacKenzie *et al.* 2002, 2006; Royle & Kéry 2007).

### Estimating current and potential distributions

A key decision in estimating suitable and unsuitable habitat is the choice of threshold (Liu *et al.* 2005). We chose to use a threshold that gave a false negative error rate of 0·05 because we had reliable information on presences but due to imperfect detection there may have been some sites where deer were present but unobserved. For invasive species it may often be desirable to minimize the false negative error. In some cases the spatial predictions of probability of suitable habitat may be more useful to managers than the demarcation into suitable and unsuitable habitat.

We used kernel smoothing to define the current range of sambar deer in Victoria. Kernel smoothing has previously been applied to the modelling of presence-only species distribution data (Hengl *et al.* 2009) but our innovation was to use the resulting distribution to delimit suitable habitat estimated from the occupancy model into occupied habitat (current range) and unoccupied habitat (potential range), a critical parameter in decision-making for invasive species (Hulme 2006). The kernel density estimator, and hence estimates of current distribution, can be particularly sensitive to the choice of smoothing parameter (Diggle 2003). As well as calculating the smoothing parameter using the robust method developed by Sheather & Jones (1991), we also used historical information on the range expansion of sambar deer in Victoria (Menkhorst 1995; Bentley 1998) to help us determine the ‘best’ model of occupied and unoccupied range.

### Management applications

The draft management policy focuses on containing invasive sambar deer within their current distribution in Victoria (Department of Sustainability and Environment 2009a). Although we have shown that sambar deer occupy *c.* 74% of their potential range in Victoria, our analysis has identified several discrete areas of suitable habitat that sambar deer do not currently occupy (Fig. 7). The Great Otway National Park and Grampians National Park are both separated from occupied range by agricultural land that sambar deer are unlikely to disperse across (Downes 1983; Bentley 1998). However, illegal translocation to establish new populations of deer has commonly occurred in Australia (Moriarty 2004). Rapid eradication of new populations has been proposed as a priority management action for sambar deer in Victoria (Department of Sustainability and Environment 2009a) and establishing surveillance monitoring in areas of suitable but unoccupied habitat using our detection methods (Fig. 4) would enable such populations to be quickly detected and dealt with.

## Conclusion

Our framework enables managers to robustly estimate the current and potential distributions of established invasive species using either presence-only and/or presence–absence data, and could be applied to any plant or animal taxa. Invasive species managers can use this information to better target control and/or containment actions within the current distribution and establish surveillance monitoring to detect incursions within the potential distribution.

## Supporting Information

**Appendix S1.** The 30 biophysical variables considered as predictor variables of sambar deer habitat suitability and occupancy.

**Appendix S2.** WinBUGS code used to fit the occupancy model to presence–absence data from field surveys.

**Table S1.** Model selection summary for the 17 models of sambar deer occupancy used for model averaging.

**Fig. S1.** Maxent output of the response of sambar deer to the four covariates that explained the most variation in the habitat suitability model of incidental sightings.

**Fig. S2.** Output of the (a) Maxent habitat suitability and (b) occupancy models rescaled as deciles.

As a service to our authors and readers, this journal provides supporting information supplied by the authors. Such materials may be re-organized for online delivery, but are not copy-edited or typeset. Technical support issues arising from supporting information (other than missing files) should be addressed to the authors.
