# Supplementary material for: Using presence-only and presence–absence data to estimate the current and potential distributions of established invasive species
Source: J Appl Ecol. 2011 Feb;48(1):25–34. doi: 10.1111/j.1365-2664.2010.01911.x (PMC3038347; doi:10.1111/j.1365-2664.2010.01911.x)
Supplement: Supplementary file 1 [file jpe0048-0025-SD1.doc]

**Appendix S1.** The 30 biophysical variables considered as predictor variables of sambar deer habitat suitability and occupancy.

All 30 GIS layers used in this study were resampled to have a pixel size of 2-km × 2-km and an equal extent covering the state of Victoria. The original source GIS layers had resolutions of either 25m (Landsat images) or 100m (Digital Elevation Model). Resampling was performed to maintain maximum information within the larger pixels: continuous values (e.g. elevation, climate variables and % area) were averaged and integer values (e.g. gullies) were summed.

The table below lists the names of the 30 layers, a brief description of each layer, the source group from which each layer was derived, and the source pixel size. Source group abbreviations are as follow: DEM, Digital Elevation Model; LLCM, Landsat Land Cover Model. Climate layers were constructed using Anuclim 5.1^1^. Further information will be provided by the authors upon request.

| Layer | Description | Source  group | Source pixel  size (m) |
| --- | --- | --- | --- |
| Elevation | Statewide Digital Elevation Model | DEM | 100 |
| Gullies | Gullies detected from DEM | DEM | 100 |
| RadGlobal | Solar Radiation Global Value | DEM | 100 |
| RiseX | Cartesian transform slope and aspect in east direction | DEM | 100 |
| RiseY | Cartesian transform slope and aspect in north direction | DEM | 100 |
| Slope | Slope derived from RiseX and RiseY | DEM | 100 |
| TWI | Terrain Wetness Index | DEM | 100 |
| ClearedLand | Percent area cleared land | LLCM | 25 |
| Dissimilarity | Dissimilarity index of Land Cover classes | LLCM | 25 |
| DistEdge | Distance to edge of patch of trees from within patch | LLCM | 25 |
| EdgeDist | Distance to edge of patch of trees from outside of patch | LLCM | 25 |
| Homogeneity | Homogeneity index of Land Cover classes | LLCM | 25 |
| IrrigatedLand2K | Percent area irrigated land | LLCM | 25 |
| Urban | Percent area urban land | LLCM | 25 |
| WetForestCover | Percent wet sclerophyll forest area | LLCM | 25 |
| Grass | Native grass present | LLCM | 25 |
| NativeGrassShrub | Percent area of native grass or shrubs | LLCM | 25 |
| NativeTree | Percent area native trees | LLCM | 25 |
| DistCoast | Distance to coastline | Polygon layers | ─ |
| RoadDistance | Distance to minor or greater road | Polygon layers | ─ |
| WaterDistance | Distance to fresh water body | Polygon layers | ─ |
| AnnEvap | Annual evaporation | Climate layers | ─ |
| AnnMeanRad | Annual mean solar radiation | Climate layers | ─ |
| MeanTemp | Annual mean temperature | Climate layers | ─ |
| AnnualPrecip | Annual mean precipitation | Climate layers | ─ |
| MaxTempWarmest | Maximum temperature warmest season | Climate layers | ─ |
| MinimumTemp | Minimum temperature coldest season | Climate layers | ─ |
| SeasonalPrecip | Precipitation driest season | Climate layers | ─ |
| PrecipSeasonality | Precipitation seasonality index | Climate layers | ─ |
| PrecipWettest | Precipitation in wettest season | Climate layers | ─ |

^1^Houlder, D.J., Hutchinson, M.F., Nix, H.A. & McMahon, J.P. (2000) *ANUCLIM User Guide Version 5.1*. Centre for Resource and Environmental Studies, Australian National University, Canberra.
