# Supplementary material for: Using presence-only and presence–absence data to estimate the current and potential distributions of established invasive species
Source: J Appl Ecol. 2011 Feb;48(1):25–34. doi: 10.1111/j.1365-2664.2010.01911.x (PMC3038347; doi:10.1111/j.1365-2664.2010.01911.x)
Supplement: Supplementary file 2 [file jpe0048-0025-SD2.doc]

**Appendix S2.** WinBUGS code used to fit the occupancy model to presence-absence data from field surveys.

#Y[i,j] is the observed presence/absence data, site i, sample j

#nsites is number of sites, ncov is number of covariates

#X[i,k] is the value of the covariate k at site i

model{

for (i in 1:nsites){

#z indicates presence/absence on surveys within a site.

#Pr(zp|z=1) = p, whilst Pr(zp|z=0) = 0.

z[i]~dbern(psi[i])

for (j in 1:t[i]){

zp[i,j]<-z[i]*p[j]

Y[i,j]~dbern(zp[i,j])

}}

#psi as a function of ncov covariates

for (i in 1:nsites){

psi[i]<-1/(1+exp(-logit.psi[i]))

logit.psi[i]<-a.int + sum(temp.b[i,])

for (k in 1:ncov){

temp.b[i,k]<-beta[k]*X[i,k]

}}

#Priors on parameters

a.int~dnorm(0,0.001)

for (k in 1:ncov){

beta[k]~dnorm(0,0.001)

}

for (j in 1:5){

p[j]<-p.method[method[j]]

}

for (k in 1:3){

p.method[k]~dunif(0,1)

}

#Functions of parameters

ztot<-sum(z[])

p.tr[1]<-1-((1-p[1]))

p.tr[2]<-1-((1-p[1])*(1-p[2]))

p.tr[3]<-1-((1-p[1])*(1-p[2])*(1-p[3]))

p.sign<-p[4]

p.cam<-1-pow((1-p[5]),0.5)

}
