# Supplementary material for: Using presence-only and presence–absence data to estimate the current and potential distributions of established invasive species
Source: J Appl Ecol. 2011 Feb;48(1):25–34. doi: 10.1111/j.1365-2664.2010.01911.x (PMC3038347; doi:10.1111/j.1365-2664.2010.01911.x)
Supplement: Supplementary file 3 [file jpe0048-0025-SD3.doc]

**Fig. S1.** Maxent output of the response of sambar deer to the four covariates that explained the most variation in the habitat suitability model of incidental sightings.
