# Supplementary figures and images for: Using presence-only and presence–absence data to estimate the current and potential distributions of established invasive species
Source: J Appl Ecol. 2011 Feb;48(1):25–34. doi: 10.1111/j.1365-2664.2010.01911.x (PMC3038347; doi:10.1111/j.1365-2664.2010.01911.x)

**Fig. S2.** Output of the (a) Maxent habitat suitability and (b) occupancy models rescaled as deciles.


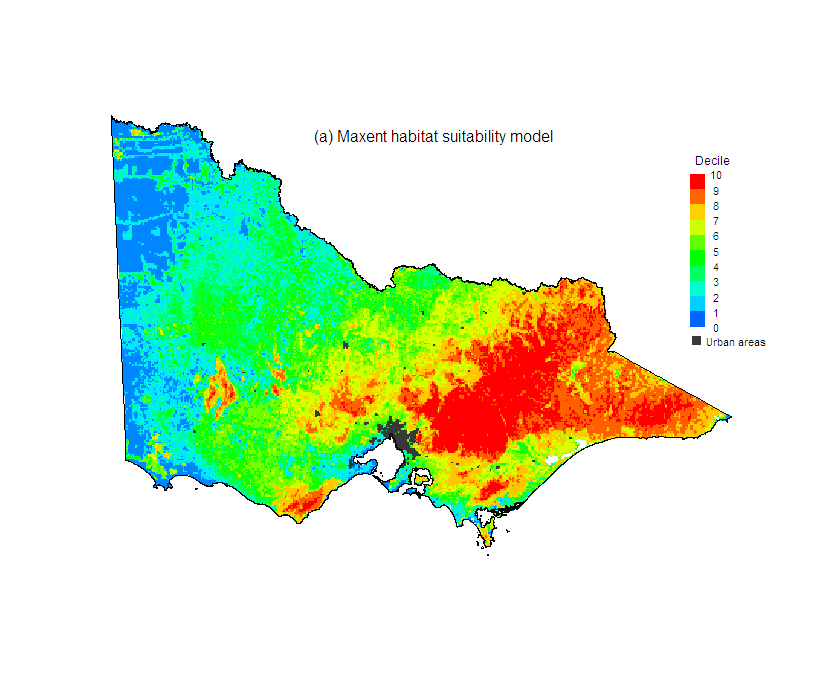


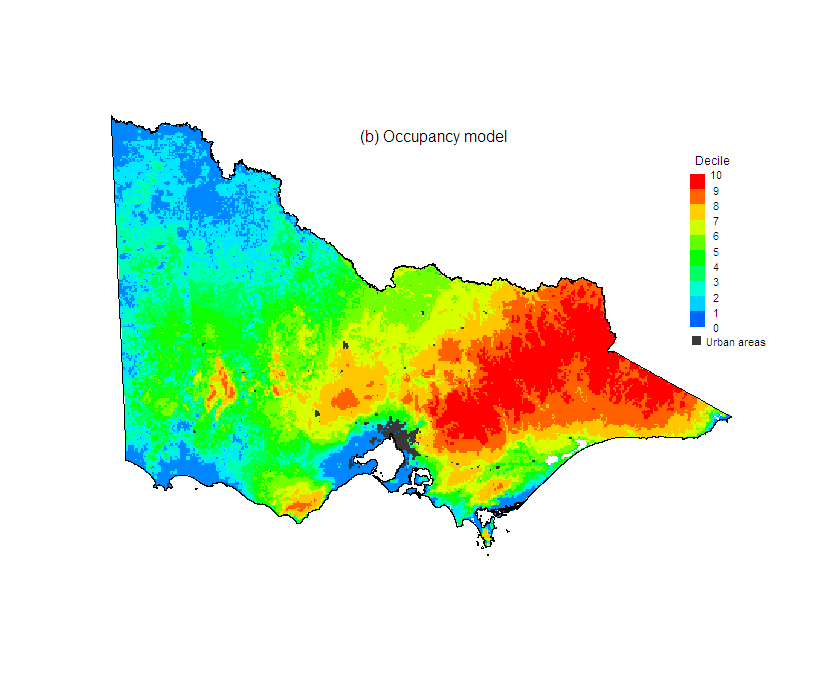

Supplement: Supplementary file 4 [file jpe0048-0025-SD4.doc]
