# Supplementary material for: Using presence-only and presence–absence data to estimate the current and potential distributions of established invasive species
Source: J Appl Ecol. 2011 Feb;48(1):25–34. doi: 10.1111/j.1365-2664.2010.01911.x (PMC3038347; doi:10.1111/j.1365-2664.2010.01911.x)
Supplement: Supplementary file 5 [file jpe0048-0025-SD5.doc]

**Table S1.** Model selection summary for the 17 models of sambar deer occupancy used for model averaging, along with DIC values, difference in DIC (∆DIC) and model weights (*w*) calculated from this reduced set of 17 models.

| Model | DIC | ∆DIC | *w* |
| --- | --- | --- | --- |
| Gullies + AnnualPrecip + MeanTemp + MinimumTemp | 258.73 | 0.00 | 0.185 |
| AnnualPrecip + MinimumTemp | 259.98 | 1.25 | 0.099 |
| AnnualPrecip + MeanTemp + MinimumTemp | 260.37 | 1.64 | 0.081 |
| Gullies + AnnualPrecip + MinimumTemp | 260.52 | 1.80 | 0.075 |
| AnnualPrecip + MinimumTemp + Slope | 260.68 | 1.95 | 0.070 |
| Homogeneity + AnnualPrecip + MinimumTemp | 260.82 | 2.09 | 0.065 |
| AnnualPrecip + MeanTemp + MinimumTemp + Slope | 260.89 | 2.17 | 0.063 |
| AnnualPrecip + MinimumTemp + WetForestCover | 261.02 | 2.29 | 0.059 |
| Homogeneity + AnnualPrecip + MinimumTemp + Slope | 261.74 | 3.01 | 0.041 |
| Gullies + AnnualPrecip + MinimumTemp + Slope | 261.77 | 3.04 | 0.040 |
| Homogeneity + AnnualPrecip + MinimumTemp + WetForestCover | 261.85 | 3.12 | 0.039 |
| AnnualPrecip + MeanTemp + MinimumTemp + WetForestCover | 261.92 | 3.19 | 0.037 |
| Homogeneity + AnnualPrecip + MeanTemp + MinimumTemp | 262.03 | 3.30 | 0.035 |
| Gullies + AnnualPrecip + MinimumTemp + WetForestCover | 262.11 | 3.38 | 0.034 |
| AnnualPrecip + MinimumTemp + WetForestCover + Slope | 262.13 | 3.40 | 0.034 |
| Gullies + Homogeneity + AnnualPrecip + MinimumTemp | 262.14 | 3.41 | 0.034 |
| MinimumTemp + WetForestCover + Slope | 264.76 | 6.03 | 0.009 |
